# Joint Meeting of the South West Radiologists Association and the Societe d'Electro-Radiologie Medicale de l'Quest, St Malo, June 1990

**Published:** 1990-09

**Authors:** Martin H. Morse


### Joint Meeting of the South West Radiologists Association and the Societé d'Electro-Radiologie Medicale de l'Ouest, St. Malo, June 1990

The South West Radiologists Association (SWRA) were the guests of the Societe d'Electro-Radiologie Medicale de l'Ouest (SERMO) for the most recent in their series of biennial joint meetings, which was held in St. Malo, Brittany, during the weekend of 8–10th June. The gathering unfortunately clashed with the British Institute of Radiology conference in Harrogate, the number of SWRA members attending was therefore somewhat less than would otherwise have been the case.

The scientific session took place at the Centre Hopitalier de St. Malo, and was attended by approximately 60 Radiologists. The 'key-note' lecture was given by Professor Rancois Joffre (Toulouse), on the place of the newer endoluminal techniques in the treatment of lower limb arterial disease. He reviewed both local and international experience in laser angioplasty, thrombolysis, stenting, atherectomy and angioscopy. There was an active discussion following his talk, with unanimous accord that the case for laser angioplasty remains firmly 'not proven'. The remainder of the day consisted of short presentations, both in English and in French, the majority of which were related to vascular diagnostic and interventional work. Biliary intervention, MRI in haemophilia, and CT in alveolar bone loss were mentioned, as was a rather worrying short series of acute subdural haematomata following lumbar puncture. SWRA presentations were made by Drs Terry Brown (Taunton, Chairman SWRA), on ultrasound in breast disease; Tim Lewis (Frenchay), on assessment of cervical fusion following Clowards operation; and Martin Morse (Frenchay), on complications in angioplasty. The session concluded with a far ranging view from Professor Michel Bellet (Brest), on the future place of Radiology in the undergraduate and general postgraduate curriculum, and provisional planning for an integrated European training scheme.

Mention must be made of the superb hospitality which we encountered. A welcoming dinner in St. Malo on Friday night was a difficult act to follow, but the main conference function on Saturday, on a balmy summers evening in the famous oyster centre of Cancale, managed it by a short head. The accompanying persons programme included a visit to Dinan, where the lunch was, according to my informant, the gastronomic 'tour de force' of the entire visit. The rest of us managed on hospital food for Saturday lunch (I must ask our catering staff why they cannot put on stuffed langoustines and individual charlottes russe more often). On Sunday morning, the full party staggered around the abbey of Mont St. Michel, faint hearts beating stronger on the descent to the world-renowned Mere Poulard restaurant where our lunch awaited us, and to which we were able to do surprisingly good justice. The din in the coach returning us to St. Malo, as further expressions of international well being were compounded by attempts at risqué jokes in Franglais and hectic plans for the next meeting (on English soil in 1992) gradually gave way to contented snoring; it was a somewhat bleary-eyed group who finally exchanged 'au-revoirs' before either driving south or sailing north, the prospect of the Monday morning reporting pile being only dimly visible on the horizon.

Particular thanks must go to the President of SERMO, Professor Ramee, and to the chairman of the organising committee, Professor Carfsin, for making us so welcome. Mrs Elizabeth King acted as our liason for the trip, and we are grateful to her for minimizing the number of setbacks to Anglo-French understanding which would undobtedly have occurred in her absence. Commiserations go to Dr Robin Sellwood (Truro, Secretary SWRA), who having successfully piloted both the SWRA end of the organisation, and his sailing boat from Falmouth to St. Malo, was stricken by a local bug and missed the Saturday evening and Sunday programmes.

In essence, a good quality scientific meeting in a delightful location, with excellent company and superb cuisine. Harrogate may have its attractions, but give me St. Malo any time!

Martin H. Morse.

